# Neck Circumference as a Predictive Indicator of CKD for High Cardiovascular Risk Patients

**DOI:** 10.1155/2015/745410

**Published:** 2015-07-29

**Authors:** Ya-Fang Liu, Shih-Tai Chang, Wei-Shiang Lin, Jen-Te Hsu, Chang-Min Chung, Jung-Jung Chang, Kuo-Chun Hung, Kang-Hua Chen, Chi-Wen Chang, Fu-Chi Chen, Yun-Wen Shih, Chi-Ming Chu

**Affiliations:** ^1^Graduate Institute of Life Sciences, National Defense Medical Center, No. 161, Section 6, Minquan East Road, Neihu District, Taipei City 114, Taiwan; ^2^Department of Education and Research, Shin Kong Wu Ho-Su Memorial Hospital, No. 95, Wenchang Road, Shilin District, Taipei City 111, Taiwan; ^3^Division of Cardiology, Chiayi Chang Gung Memorial Hospital, No. 6, West Section Chiapu Road, Putzu City, Chiayi Hsien 613, Taiwan; ^4^Division of Cardiology, Department of Medicine, Tri-Service General Hospital, National Defense Medical Center, No. 325, Section 2, Chenggong Road, Neihu District, Taipei City 114, Taiwan; ^5^Division of Cardiology, Department of Internal Medicine, Chang Gung Memorial Hospital, Chang Gung University College of Medicine, No. 5, Fuxing Street, Guishan District, Taoyuan City 333, Taiwan; ^6^School of Nursing, College of Medicine, Chang Gung University, No. 259, Wenhua 1st Road, Guishan District, Taoyuan City 333, Taiwan; ^7^Department of Biomedical Engineering, National Defense Medical Center, No. 161, Section 6, Minquan East Road, Neihu District, Taipei City 114, Taiwan; ^8^Section of Biostatistics and Informatics, Department of Epidemiology, School of Public Health, National Defense Medical Center, Room 4317, No. 161, Section 6, Minquan East Road, Neihu District, Taipei City 114, Taiwan

## Abstract

*Background*. Neck circumference (NC) is an anthropometric measure of obesity for upper subcutaneous adipose tissue distribution which is associated with cardiometabolic risk. This study investigated whether NC is associated with indicators of chronic kidney disease (CKD) for high cardiometabolic risk patients. *Methods*. A total of 177 consecutive patients who underwent the outpatient departments of cardiology were prospectively enrolled in the study. The patients were aged >20 years with normal renal function or with stages 1–4 CKD. A linear regression was performed using the Enter method to present an unadjusted *R* ^2^, standardized coefficients, and standard error, and the Durbin-Watson test was used to assess residual independence. *Results*. Most anthropometric measurements from patients aged ≧65 were lower than those from patients aged <65, except for women's waist circumference (WC) and waist hip ratio. Female NC obtained the highest *R* ^2^ values for 24 hr CCR, uric acid, microalbuminuria, hsCRP, triglycerides, and HDL compared to BMI, WC, and hip circumference. The significances of female NC with 24 hr CCR and uric acid were improved after adjusted age and serum creatinine. *Conclusions*. NC is associated with indicators of CKD for high cardiometabolic risk patients and can be routinely measured as easy as WC in the future.

## 1. Introduction

Cardiovascular disease (CVD) and chronic kidney disease (CKD) are two important global public health concerns that share common cardiometabolic risk factors that include hypertension, diabetes mellitus, and hyperlipidemia. The above three diseases generally coexist, making them the most common risk factors for CVD [1]. In 2007, the Taiwan Health Promotion Administration of the Ministry of Health and Welfare conducted a survey involving 5,895 healthy individuals in Taiwan and concluded that, among those patients with hypertension, diabetes mellitus, and hyperlipidemia, their rates of developing heart disease in 5 years are 1.9, 1.5, and 1.8 times greater than those without hypertension, diabetes mellitus, and hyperlipidemia, respectively [2]. Cardiovascular events include fatal and nonfatal cardiovascular events (stroke, infarction, angina, and heart failure). Hypertension, diabetes mellitus, and hyperlipidemia contribute to the increased risk of experiencing cardiovascular events [3]. It is suggested that patients who currently have CVD or have received coronary revascularization should take steps to mitigate their cardiovascular risks [4].

Hypertension, diabetes mellitus, and hyperlipidemia are also initiation factors for CKD [5]. One study showed that mild renal insufficiency was common among community-based citizens and that this condition was related to the high prevalence of CVD. Mild renal insufficiency increases the risk of cardiovascular events and total mortality [6]. It was reported that a lower estimated glomerular filtration rate (eGFR) correlates with a higher risk of cardiovascular events and the related death [7]. Microvascular abnormality is a common triggering factor for both CVD and CKD [8]. The Kidney Early Evaluation Program study suggested that conditions for cardiovascular risk are defined as a urine albumin: creatinine ratio ≥30 mg/g (3.4 mg/mmol) or an eGFR < 60 mL/min/1.73 m^2^ [7].

The main complication of CKD is CVD. Observations made in the Kidney Early Evaluation Program and the National Health and Nutrition Examination Survey studies suggested that CKD may increase the prevalence of myocardial infarction or stroke [8]. Medium to severe renal function impairments exacerbate atherosclerosis, which in turn leads to an increased risk of cardiovascular events [9]. This means the deaths of CKD patients are more likely caused by CVD than by progression of kidney failure [10]. However, patients with CVD also suffer from a higher risk of CKD [11]. Patients who have undergone coronary revascularization must be aware of their potential CKD risk for the future. That CKD may also cause atherosclerosis in coronary arteries and the cerebrovascular and peripheral circulatory system [12]. Additionally, the coexistence of CVD and CKD leads to higher mortality [6]. Therefore, CVD and CKD should be considered mutual risk factors for each other. Among CVD patients the CKD prevention is necessary, and similar condition is conversely alike. Therefore, development of an indicator that predicts the risk of both conditions would help to effectively treat the coexistence of both diseases.

Abdominal obesity and general obesity would cause increases in triglycerides, low density lipoprotein (LDL) cholesterol, blood sugar, insulin, and blood pressures. They will also cause decreases in high density lipoprotein (HDL) cholesterol. All of these factors may lead to onset of CVD, which in turn increases coronary heart disease onset or mortality and total mortality [13, 14]. Neck circumference (NC) is an indicator of upper body fat distribution, whereas body morphologies and fat distribution can be used as factors to assess the risk of obesity [15]. In addition to body mass index (BMI), waist circumference, and hip circumference measurements as indicators of obesity, NC can also be used as a novel indicator for cardiometabolic risk [16, 17]. However, previous studies have not focused on NC with CKD. This pilot study aimed to investigate the relationship between NC and CKD. We intend to confirm whether NC is as an associated indicator of CKD for high cardiovascular risk patients or whether it is as an alternative option for disabled or bedbound patients for whom other body anthropometric indicator measurements are not suitable.

## 2. Materials and Methods

### 2.1. Research Framework and Participants

**Figure 1:**
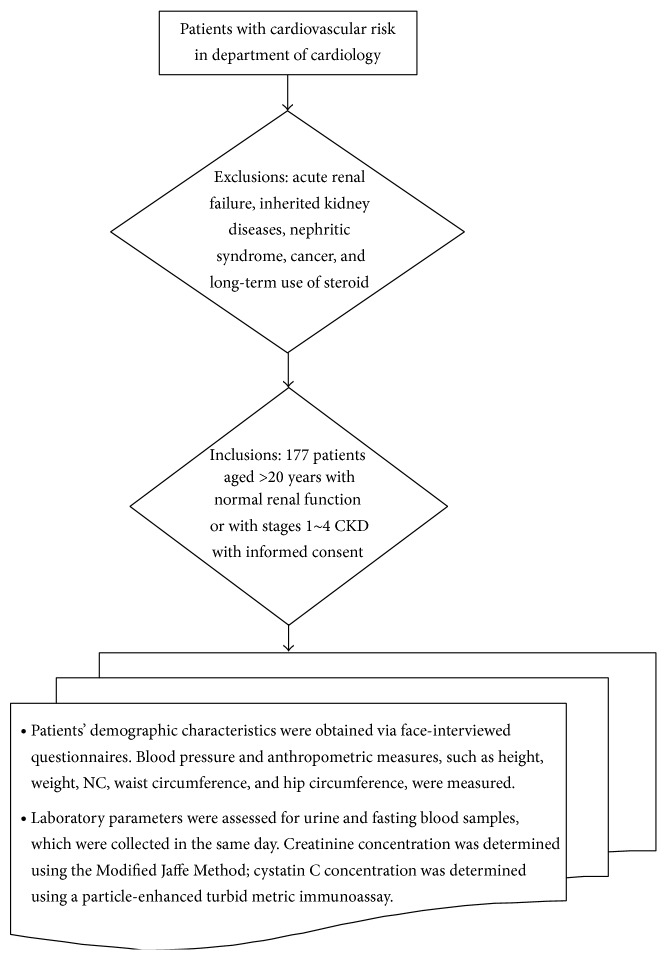
Research framework and participants.

This study, as given in Figure 1, involved 177 patients with cardiovascular risk. Inclusion criteria were patients aged >20 years with normal renal function or with stages 1~4 CKD, excluding conditions such as acute renal failure, inherited kidney diseases, nephritic syndrome, cancer, and long-term use of steroid. This study was approved by the Institute Review Committees at Tri-Service General Hospital and Chiayi Chang Gung Memorial Hospital and all the participants signed informed consent prior to study enrolment.

### 2.2. Data Collection

Patients' demographic characteristics were obtained via face-interviewed questionnaires. Blood pressure and anthropometric measures, such as height, weight, NC, waist circumference, and hip circumference, were measured. Laboratory parameters were assessed for urine and fasting blood samples, which were collected in the same day. Creatinine concentration was determined using the Modified Jaffe Method with a SYNCHRON LXI 725 (Tokyo, Japan) instrument; cystatin C concentration was determined using a particle-enhanced turbid metric immunoassay with a Hitachi 7170 (Tokyo, Japan) instrument.

### 2.3. Assessment of Renal Function

GFR was calculated based on the 24-hour urine creatinine clearance rate (24 hr CCR) to assess renal function. The equation for 24 hr CCR is as follows: (24 hr urine total volume × urine creatinine concentration)/(creatinine concentration × 1440). The eGFR calculation was based on Modification of Diet in Renal Disease (MDRD), as eGFR_MDRD_ = 186 × crea^−1.154^ × age^−0.203^ × (0.742 if female) × (1.212 if black), and Cockcroft-Gault Equation (CG), as eGFR_CG_ = [(140 − age) × weight (kg)] ÷ (creatinine (mg/dL) × 72) × (0.85 if female).

### 2.4. Data Processing

All data were grouped by gender or age. A *t*-test was performed to assess the differences between ages within the same gender groups. The relevance between NC and each laboratory measurement was assessed using the Spearman correlation analysis. Continuous data for males and females were analyzed separately using the Kolmogorov-Smirnov test or the Shapiro-Wilk test for normal distribution. If data were not normally distributed, logarithmic transformation of the data was performed to make the data fit a normal distribution. To investigate the relevance between anthropometric measures and heart disease and CKD risk factors, a linear regression was performed using the Enter method to present an unadjusted *R* ^2^, standardized coefficients, and standard error, and the Durbin-Watson test was used to assess residual independence. An age adjustment was made for all assessed cardiovascular risk factors. To avoid the impact of renal function differences in individuals, age and log(creatinine) were included to correct the risk factors assessment. All statistical analyses were performed using SPSS 18.0 (Statistical Package for Social Sciences, Chicago, IL, USA). A *P* value < 0.05 was considered a statistically significant level.

## 3. Results

The characteristics of 177 participants in this study are presented in Table 1. There were 122 (71%) males with a mean age of 66 and 50 (29%) females with a mean age of 67. For most anthropometric measures, the measurements from patients ≧ 65 years old were lower than those from patients < 65 years old, except for women's waist circumference and waist hip ratio. Differences were observed in weight, BMI, diastolic blood pressure, triglycerides, serum creatinine, cystatin C, phosphorus, 24 hr CCR, eGFR_MDRD_, and eGFR_CG_ between different ages among males. In each age of females, the differences were observed in height, weight, BMI, hip circumference, waist hip ratio, NC, cystatin C, albumin, urine creatinine, 24 hr CCR, and eGFR_CG_. Elderly patients of both genders typically perform a poor renal function.

Table 2 showed the Spearman correlation coefficients with NC between both sexes. In males, most relevant coefficients were significant, especially those anthropometric measures, fasting glucose, triglycerides, cystatin C, aspartate aminotransferase, 24 hr CCR, and eGFR_CG_, where positive correlations were observed between NC and 24 hr CCR, eGFR_CG_, hsCRP, triglycerides, and LDL cholesterol. In females, diastolic blood pressure, triglycerides, uric acid, albumin, high-sensitivity C-reactive protein (hsCRP), total protein, phosphorus, and eGFR_CG_ reached significance, where negative correlations were observed between NC and total cholesterol, HDL, total protein, aspartate aminotransferase, alanine aminotransferase, phosphorus, sodium, and potassium.

Regression curves after age adjustment are shown in Table 3(a). Compared to BMI, waist circumference, and hip circumference, the higher *R* ^2^ values were obtained for female NC and microalbuminuria, hsCRP, triglycerides, and HDL cholesterol. Age- and log(creatinine)-adjusted curves are shown in Table 3(b). The items showing statistical significance in male and female NC are roughly the same as those in Table 3(a). Female NC was adjusted for age and log(creatinine), the significance of 24 hr CCR and uric acid was improved. Compared to BMI, waist circumference, and hip circumference, the highest *R* ^2^ values were obtained for female NC and 24 hr CCR, uric acid, microalbuminuria, hsCRP, triglycerides, and HDL cholesterol (negative correlation).

## 4. Discussion

Several studies have revealed the relationship between body adipose abnormalities, CVD, and metabolic syndrome [16–29] as well as between cardiometabolic factors and CKD [1, 4, 5, 7–11, 30, 31]. However, there is lack of reporting on the relevance between NC and CKD. This is the first study to report their relationship and it is discovered that NC is associated with indicators of renal disease such as 24 hr CCR, eGFR_CG_, uric acid, and urine microalbuminuria, in addition to conventional cardiovascular risk factors such as hsCRP, triglycerides, LDL cholesterol, and HDL cholesterol.

NC is an alternative measurement for upper-body subcutaneous fat, and therefore NC may play a vital role in CVD clinical prediction [16]. NC as an associated factor for diabetes, after adjusted BMI and waist circumference, was the only risk factor related to type II diabetes mellitus [26]. These results confirmed that NC measurements can be used as an effective clinical screening tool for insulin resistance, and can be used as powerful indicators to improve the screening ability for type II diabetes mellitus. In addition to insulin resistance and type II diabetes mellitus, NC also has associated power to assess cardiometabolic risk [28].

NC also correlated significantly with intima-media thickness of common or internal carotid arteries after BMI and waist circumference adjustment. For every 1-standard deviation unit increase in NC, there is a 0.025 mm thickness increase in common carotid artery, and remaining significant even after BMI adjustment [27]. In a follow-up study involving acute ischemic stroke patients who have a 1-year total mortality of 8.9%, the author discovered that aging and larger neck circumference were more frequent findings among the dead, but not obesity. Therefore, NC is a critical clinical warning factor for fatal outcomes in acute ischemic stroke [25]. Insulin resistance, related with NC, causes arterial stiffness, which in turn has an impact on CKD and even nondiabetic CKD. CKD patients who develop metabolic syndrome would also have a higher risk of arterial stiffness [30].

Although gender differences of anthropometric measures, systolic blood pressure, total cholesterol, LDL cholesterol, HDL cholesterol, and fasting plasma glucose exist, NC is also related to cardiometabolic risk [16] and is a powerful indicator for predicting dyslipidemia. In particular, the correlation between triglycerides and NC is stronger than BMI and waist circumference [28]. Moreover, the relevance and importance of waist circumference and metabolic syndrome to CVD have been discussed previously [32]. One study showed that dual indicators using both triglycerides and waist circumference can assess the risk of coronary artery disease in patients with abdominal obesity. When the Framingham model was used for predicting CVD risk, these dual indicators may help to add associated value. The hypertriglyceridemic-waist phenotype might become a superior health indicator compared to metabolic syndrome [33]. Another study in CKD patients suggested that the hypertriglyceridemic-waist phenotype can be used to evaluate the severity of carotid atherosclerosis and can also be used as an effective indicator to predict CVD risk [31]. Our study showed NC is highly correlated to waist circumference and triglycerides. Therefore, NC may replace waist circumference for phenotyping, especially because this phenotype can be used for both coronary heart disease and CKD. NC is an ideal associated indicator for these two coexisting diseases that are risk factors for each other. Moreover, for patients who are disabled, in serious condition, in particular those who are bedridden, or even in healthy status, this method would be not only simple but also easy to perform with high efficiency and convenience. Therefore, NC may be popularized for clinical applications as an associated indicator of risk for both outcomes.

Based on the eGFR_CG_ equation, we know that weight and eGFR are positively correlated. Specifically, heavier weights correlate with higher eGFR_CG_ among those with the same sex, age, and creatinine level. From Table 2 in this study, we can conclude that NC and weight are positively correlated. Subjects aged <65 years have higher mean NC values and also have higher mean eGFR_CG_ values and eGFR_MDRD_, indicating that patients with higher NC values usually have better renal function. This means that NC is positively correlated with eGFR. The eGFR_MDRD_ and eGFR_CG_ equations had the gender-specific coefficient of creatinine.

Hoebel et al. have established the cutoffs for NC to evaluate the risk of metabolic syndrome, which were 40 cm and 41 cm for the young and older men, 34 cm and 33 cm for young and older women, respectively [22], which indicates potential uses of NC for other purposes. Body morphologies, other than NC, have an existing normal level; out of which indicate unhealthy conditions. Appropriate cut-off values for NC should be established, in the future, to assess early stage risk of CKD. This cross-sectional study relied on hospital-based samples. It is suggested that a future population-based study and a cohort-based study with follow-up should be conducted, so that the importance of NC to the CKD risk prediction can be validated, and it can be determined whether this importance will change with increasing age. However, in fashion experience and healthy population, the circumference of someone's neck would be about the same as his/her pants width; that is, the waist circumference is about twice the neck circumference [34]. Several studies report similar ratios around 1.9 to 2.1 for circumferences of waist to neck in control groups [16, 17, 21, 22, 29].

Many indicators are testing for obesity and NC is an anthropometric measure of obesity for upper subcutaneous adipose tissue distribution which is associated with cardiometabolic risk, while NC is an alternative and novel indicator to test body fat distribution [17], and it is also a simple and time-saving screening tool to capture overweight or obese patient [20]. NC also is an easy associated tool for metabolic syndrome and insulin resistance [22] and a powerful indicator of atherosclerotic lipid abnormalities and their risk factors [17]. NC is also an independent associated factor for cardiometabolic risk [16, 29] and an effective screening test for cardiovascular risk in children [18] or even in obese children [23]. Studies mentioned above have suggested that NC is a superior indicator for metabolic syndrome in terms of both relevance and significance compared to BMI and waist circumference [17, 19, 21, 24, 28, 35, 36].

## 5. Conclusions

NC is associated with indicators of CKD for high cardiometabolic risk patients and can be routinely measured as easy as WC in the future.

## Figures and Tables

**Table 1:** Study characteristics by sex and age.

Variables, mean (SD)	Men, *n* = 125			Women, *n* = 52		
	<65 yrs (*n* = 57)	≧65 yrs (*n* = 68)	*P* value	<65 yrs (*n* = 26)	≧65 yrs (*n* = 26)	*P* value
Age (yrs)	58 (5.2)	73.7 (5.3)	<0.001	58.6 (5.5)	75.1 (6)	<0.001
Height (cm)	166.2 (6.1)	164.8 (4.8)	0.166	157 (5.7)	153 (5.8)	0.015
Weight (kg)	76.8 (11.8)	70.8 (10.4)	0.003	68.9 (12)	59.7 (8.6)	0.002
Body mass index (kg/m^2^)	27.7 (3.5)	26 (3.7)	0.011	27.9 (4)	25.5 (3)	0.017
Waist circumference (cm)	97.3 (9.7)	94.5 (9.3)	0.104	92.3 (11.3)	95.8 (9.2)	0.230
Hip circumference (cm)	101.8 (7.2)	100.2 (7.2)	0.224	102.3 (10.4)	97.5 (5.9)	0.049
Waist hip ratio	1 (0.1)	0.9 (0.1)	0.198	0.9 (0.1)	1 (0.1)	0.001
Neck circumference (cm)	40.6 (3.3)	39.6 (3.3)	0.100	36.6 (2.9)	34.7 (2.6)	0.017
Systolic blood pressure (mmHg)	137.3 (12.3)	136.4 (16.1)	0.732	132.1 (12)	133.9 (11.7)	0.588
Diastolic blood pressure (mmHg)	79.9 (6.7)	76.8 (8.9)	0.030	78 (8)	73.9 (8.3)	0.073
Fasting glucose (mg/dL)	119.5 (44.2)	110.9 (27)	0.205	111.3 (30.5)	112.5 (27.6)	0.883
Total cholesterol (mg/dL)	183.2 (34.5)	181.2 (32.2)	0.748	205.6 (41.1)	197.8 (29.4)	0.448
LDL cholesterol (mg/dL)	114.5 (29.9)	114.5 (27.1)	0.990	123.5 (37.1)	112.9 (27.4)	0.258
HDL cholesterol (mg/dL)	44.3 (7.8)	46.5 (15.4)	0.315	53.9 (12.9)	49.9 (11.3)	0.258
Triglyceride (mg/dL)	148.5 (74.4)	117.1 (46.9)	0.006	137.9 (80.1)	136.3 (53.4)	0.937
Serum creatinine (mg/dL)	1.1 (0.3)	1.3 (0.5)	0.011	0.9 (0.2)	1 (0.4)	0.584
Cystatin C (mg/L)	0.9 (0.2)	1.2 (0.4)	<0.001	0.9 (0.2)	1.1 (0.3)	0.024
Blood urea nitrogen (mg/dL)	17.9 (6.3)	20.2 (7.2)	0.075	16.9 (4.8)	18.9 (10.1)	0.373
Uric acid (mg/dL)	7 (1.6)	6.9 (1.4)	0.908	6 (1.6)	6.2 (1.5)	0.632
Albumin (mg/dL)	4.4 (0.5)	4.4 (0.3)	0.437	4.4 (0.3)	4.3 (0.2)	0.046
hsCRP (mg/L)	2 (2)	1.6 (2.2)	0.319	2.1 (2.5)	2.1 (2.7)	0.992
Microalbuminuria (mg/day)	23.4 (33)	29.5 (61.1)	0.505	29.2 (54.8)	17.6 (34.6)	0.377
Total protein (mg/dL)	7.4 (0.5)	7.6 (0.5)	0.094	7.7 (0.5)	7.5 (0.4)	0.138
Aspartate aminotransferase (U/L)	28.9 (23.1)	30.7 (30.2)	0.721	25.4 (10.6)	26.3 (20.3)	0.842
Alanine aminotransferase (U/L)	35.7 (31.2)	33.8 (37.4)	0.753	35.7 (30.9)	23 (18.7)	0.085
Alkaline phosphatase (U/L)	63.6 (24.4)	61.4 (15.1)	0.777	65.7 (15.1)	64 (33.1)	0.912
Calcium (mg/dL)	8.8 (0.5)	8.7 (0.5)	0.655	8.7 (0.5)	8.8 (0.7)	0.547
Phosphorus (mmol/L)	3.5 (0.4)	3.2 (0.5)	0.012	3.8 (0.3)	3.7 (0.5)	0.175
Sodium (mmol/L)	143.7 (2)	143.2 (2.2)	0.200	144.4 (2)	143.8 (2)	0.299
Potassium (mEq/L)	4.9 (5.3)	4.9 (5.4)	0.953	5.8 (8.1)	6.4 (9.1)	0.842
Bilirubin (mg/dL)	1.1 (1.4)	0.9 (0.5)	0.233	0.8 (0.2)	0.8 (0.3)	0.194
Urine creatinine (mg/dL)	74.6 (28.7)	79.7 (36.2)	0.395	71.9 (30.9)	56.1 (17.9)	0.034
24 hr CCR (mL/min)	103.3 (33.1)	78.1 (31.7)	<0.001	93 (33.3)	67.8 (25.9)	0.004
eGFR_MDRD_ (mL/min/1.73 m^2^)	75.1 (18.9)	62.1 (17.5)	<0.001	72.6 (20)	67 (19)	0.319
eGFR_CG_ (mL/min/1.73 m^2^)	81.7 (23.6)	53.7 (16.5)	<0.001	76.8 (23.8)	52 (15.2)	<0.001

**Table 2:** Spearman correlation coefficients of neck circumference by sex and age.

Variables	Men < 65 yrs (*n* = 57)		Men ≧ 65 yrs (*n* = 68)		Women < 65 yrs (*n* = 26)		Women ≧ 65 yrs (*n* = 26)	
	Coefficients	*P* value	Coefficients	*P* value	Coefficients	*P* value	Coefficients	*P* value
Age (yrs)	−0.263	0.048	−0.113	0.361	−0.373	0.060	0.167	0.414
Height (cm)	0.125	0.355	0.149	0.224	0.552	0.003	0.255	0.209
Weight (kg)	0.677	<0.001	0.649	<0.001	0.635	<0.001	0.760	<0.001
Body mass index (kg/m^2^)	0.738	<0.001	0.643	<0.001	0.417	0.034	0.668	<0.001
Waist circumference (cm)	0.665	<0.001	0.712	<0.001	0.331	0.098	0.622	0.001
Hip circumference (cm)	0.660	<0.001	0.743	<0.001	0.560	0.003	0.832	<0.001
Waist hip ratio	0.342	0.009	0.337	0.005	−0.054	0.794	0.114	0.579
Systolic blood pressure (mmHg)	0.059	0.665	0.230	0.059	0.159	0.438	0.185	0.365
Diastolic blood pressure (mmHg)	0.141	0.295	0.229	0.060	0.648	<0.001	−0.170	0.406
Fasting glucose (mg/dL)	0.125	0.356	0.339	0.006	−0.027	0.899	0.092	0.662
Total cholesterol (mg/dL)	0.222	0.097	0.104	0.409	−0.170	0.418	−0.032	0.879
LDL cholesterol (mg/dL)	0.227	0.089	0.223	0.074	−0.229	0.271	0.193	0.356
HDL cholesterol (mg/dL)	0.099	0.464	−0.181	0.156	−0.274	0.185	−0.261	0.218
Triglyceride (mg/dL)	0.176	0.191	0.339	0.006	0.295	0.152	0.423	0.044
Creatinine (mg/dL)	−0.167	0.215	−0.076	0.546	0.057	0.788	−0.036	0.866
Cystatin C (mg/L)	0.303	0.034	−0.100	0.450	0.276	0.226	−0.041	0.863
Blood urea nitrogen (mg/dL)	−0.040	0.773	0.116	0.377	−0.090	0.668	0.318	0.121
Uric acid (mg/dL)	−0.202	0.135	0.113	0.374	0.630	0.001	0.140	0.505
Albumin (mg/dL)	0.057	0.674	0.082	0.521	0.268	0.195	−0.397	0.050
hsCRP (mg/L)	0.188	0.174	0.244	0.058	0.627	0.001	0.053	0.806
Microalbuminuria (mg/day)	0.238	0.077	0.049	0.709	0.380	0.067	0.256	0.217
Total protein (mg/dL)	0.044	0.745	0.078	0.541	−0.040	0.849	−0.607	0.001
Aspartate aminotransferase (U/L)	0.003	0.982	−0.314	0.012	−0.011	0.957	−0.161	0.443
Alanine aminotransferase (U/L)	0.028	0.834	0.089	0.482	−0.020	0.925	−0.044	0.833
Alkaline phosphatase (U/L)	0.443	0.172	0.185	0.526	−0.319	0.538	0.559	0.192
Calcium (mg/dL)	−0.018	0.895	0.157	0.215	0.260	0.209	0.171	0.414
Phosphorus (mmol/L)	−0.121	0.373	−0.013	0.918	−0.221	0.288	−0.432	0.031
Sodium (mmol/L)	0.070	0.606	0.117	0.357	−0.134	0.524	−0.237	0.255
Potassium (mEq/L)	0.001	0.995	0.261	0.067	−0.055	0.818	−0.026	0.915
Bilirubin (mg/dL)	0.001	0.997	−0.041	0.746	0.277	0.180	−0.116	0.581
Urine creatinine (mg/dL)	0.165	0.220	0.037	0.770	0.249	0.230	−0.011	0.958
24 hr CCR (mL/min)	0.440	0.001	0.296	0.017	0.231	0.267	0.064	0.760
eGFR_MDRD_ (mL/min/1.73 m^2^)	0.216	0.107	0.077	0.541	−0.066	0.752	0.039	0.852
eGFR_CG_ (mL/min/1.73 m^2^)	0.475	<0.001	0.355	0.004	0.341	0.095	0.442	0.027

**(a):** 

Dependent variables	Men (*n* = 125)			Women (*n* = 52)		
Independent variables	*R* ^2^	*B* (SE)	*P* value	*R* ^2^	*B* (SE)	*P* value
24 hr CCR						
log⁡(Neck circumference)	0.279	269.32 (76.68)	0.001	0.232	114.479 (122.29)	0.243
log⁡(Body mass index)	0.290	181.14 (47.90)	<0.001	0.211	−26.755 (73.81)	0.719
log⁡(Waist circumference)	0.310	268.82 (62.92)	<0.001	0.210	−13.095 (87.87)	0.882
log⁡(Hip circumference)	0.337	412.97 (84.67)	<0.001	0.210	−30.973 (117.5)	0.793
eGFR_CG_						
log⁡(Neck circumference)	0.524	206.02 (44.09)	<0.001	0.503	163.779 (71.69)	0.027
log⁡(Body mass index)	0.591	172.49 (25.72)	<0.001	0.534	121.746 (41.36)	0.005
log⁡(Waist circumference)	0.601	237.27 (33.85)	<0.001	0.531	142.509 (49.32)	0.006
log⁡(Hip circumference)	0.588	312.44 (47.22)	<0.001	0.576	236.87 (62.72)	<0.001
eGFR_MDRD_						
log⁡(Neck circumference)	0.140	20.87 (46.60)	0.655	0.081	−50.119 (81.36)	0.541
log⁡(Body mass index)	0.144	25.1 (29.26)	0.393	0.130	−82.537 (47.14)	0.086
log⁡(Waist circumference)	0.150	49.83 (38.86)	0.202	0.084	−42.682 (57.52)	0.462
log⁡(Hip circumference)	0.157	85.06 (53.13)	0.112	0.096	−82.797 (76.46)	0.284
log⁡(Cystatin C)						
log⁡(Neck circumference)	0.179	0.43 (0.31)	0.166	0.210	0.131 (0.49)	0.789
log⁡(Body mass index)	0.166	−0.09 (0.19)	0.624	0.313	0.635 (0.26)	0.021
log⁡(Waist circumference)	0.166	0.15 (0.26)	0.571	0.263	0.543 (0.33)	0.104
log⁡(Hip circumference)	0.169	0.29 (0.36)	0.419	0.254	0.634 (0.42)	0.139
Uric Acid						
log⁡(Neck circumference)	0.009	−3.75 (3.99)	0.349	0.188	19.258 (6.05)	0.003
log⁡(Body mass index)	0.002	0.02 (2.45)	0.993	0.216	12.354 (3.54)	0.001
log⁡(Waist circumference)	0.002	−0.41 (3.28)	0.901	0.111	10.223 (4.48)	0.027
log⁡(Hip circumference)	0.014	−5.39 (4.48)	0.231	0.149	16.098 (5.87)	0.009
log⁡(Urine microalbuminuria)						
log⁡(Neck circumference)	0.021	2.36 (1.51)	0.120	0.200	7.255 (2.16)	0.002
log⁡(Body mass index)	0.093	3.14 (0.92)	0.001	0.105	3.089 (1.35)	0.027
log⁡(Waist circumference)	0.058	3.26 (1.23)	0.009	0.110	3.764 (1.6)	0.023
log⁡(Hip circumference)	0.049	4.09 (1.70)	0.017	0.06	3.661 (2.19)	0.102
log⁡(hsCRP)						
log⁡(Neck circumference)	0.048	2.98 (1.40)	0.036	0.101	4.366 (1.96)	0.031
log⁡(Body mass index)	0.062	2.13 (0.85)	0.014	0.067	2.091 (1.18)	0.084
log⁡(Waist circumference)	0.055	2.66 (1.14)	0.022	0.063	2.407 (1.41)	0.094
log⁡(Hip circumference)	0.043	3.15 (1.59)	0.049	0.095	3.995 (1.86)	0.037
log⁡(Systolic blood pressure)						
log⁡(Neck circumference)	0.015	0.14 (0.12)	0.252	0.025	0.175 (0.16)	0.292
log⁡(Body mass index)	0.042	0.16 (0.07)	0.031	0.003	−0.006 (0.1)	0.951
log⁡(Waist circumference)	0.031	0.18 (0.10)	0.068	0.006	0.049 (0.12)	0.674
log⁡(Hip circumference)	0.036	0.27 (0.14)	0.047	0.006	0.061 (0.16)	0.698
Diastolic blood pressure						
log⁡(Neck circumference)	0.105	27.44 (19.94)	0.171	0.260	31.523 (30.9)	0.313
log⁡(Body mass index)	0.157	37.4 (12.09)	0.002	0.045	−4.045 (18.69)	0.830
log⁡(Waist circumference)	0.114	29.63 (16.58)	0.076	0.273	29.645 (21.42)	0.173
log⁡(Hip circumference)	0.123	48.09 (22.66)	0.036	0.260	29.157 (29.33)	0.325
log⁡(Fasting plasma glucose)						
log⁡(Neck circumference)	0.023	0.48 (0.29)	0.100	0.013	0.142 (0.41)	0.731
log⁡(Body mass index)	0.055	0.47 (0.18)	0.010	0.022	0.176 (0.24)	0.474
log⁡(Waist circumference)	0.094	0.82 (0.23)	0.001	0.017	0.165 (0.29)	0.573
log⁡(Hip circumference)	0.034	0.68 (0.33)	0.042	0.016	−0.19 (0.39)	0.627
log⁡(Triglyceride)						
log⁡(Neck circumference)	0.137	1.49 (0.46)	0.002	0.172	2.45 (0.82)	0.005
log⁡(Body mass index)	0.089	0.56 (0.30)	0.064	0.145	1.379 (0.51)	0.010
log⁡(Waist circumference)	0.087	0.73 (0.40)	0.070	0.138	1.562 (0.6)	0.012
log⁡(Hip circumference)	0.094	1.12 (0.55)	0.042	0.094	1.691 (0.82)	0.044
Total cholesterol						
log⁡(Neck circumference)	0.023	134.99 (85.59)	0.117	0.007	−69.724 (153.93)	0.653
log⁡(Body mass index)	0.015	66.37 (54.09)	0.222	0.004	23.785 (91.82)	0.797
log⁡(Waist circumference)	0.012	76.58 (72.20)	0.291	0.006	46.529 (109.06)	0.672
log⁡(Hip circumference)	0.007	71.37 (99.35)	0.474	0.003	30.962 (146.12)	0.833
LDL cholesterol						
log⁡(Neck circumference)	0.040	158.88 (72.59)	0.031	0.012	27.354 (141.34)	0.847
log⁡(Body mass index)	0.048	109.69 (45.5)	0.017	0.030	80.653 (83.39)	0.338
log⁡(Waist circumference)	0.023	99.41 (61.43)	0.108	0.017	51.923 (99.87)	0.606
log⁡(Hip circumference)	0.009	84.18 (84.89)	0.323	0.016	66.655 (133.64)	0.620
log⁡(HDL cholesterol)						
log⁡(Neck circumference)	0.008	−0.19 (0.29)	0.506	0.101	−0.936 (0.42)	0.032
log⁡(Body mass index)	0.032	−0.33 (0.18)	0.071	0.023	−0.241 (0.26)	0.365
log⁡(Waist circumference)	0.011	−0.22 (0.24)	0.376	0.024	−0.294 (0.31)	0.354
log⁡(Hip circumference)	0.005	−0.14 (0.34)	0.688	0.017	−0.311 (0.43)	0.471

*R* ^2^: unadjusted *R* ^2^; *B*: unstandardized coefficients beta; SE: unstandardized coefficients Std. Error.

**(b):** 

Dependent variables	Men (*n* = 125)			Women (*n* = 52)		
Independent variables	*R* ^2^	*B* (SE)	*P* value	*R* ^2^	*B* (SE)	*P* value
24 hr CCR						
log⁡(Neck circumference)	0.642	260.86 (54.29)	<0.001	0.340	169.5 (114.94)	0.147
log⁡(Body mass index)	0.621	138.8 (35.39)	<0.001	0.310	22.78 (72.38)	0.754
log⁡(Waist circumference)	0.643	221.38 (45.7)	<0.001	0.310	17.76 (83.87)	0.833
log⁡(Hip circumference)	0.659	337.81 (61.38)	<0.001	0.309	7.43 (112.11)	0.947
eGFR_CG_						
log⁡(Neck circumference)	0.867	200.2 (23.38)	<0.001	0.800	193.98 (46.15)	<0.001
log⁡(Body mass index)	0.891	143.99 (13.43)	<0.001	0.924	193.43 (17.47)	<0.001
log⁡(Waist circumference)	0.907	205.1 (16.53)	<0.001	0.857	183.11 (27.77)	<0.001
log⁡(Hip circumference)	0.889	261.02 (24.76)	<0.001	0.910	288.32 (29.52)	<0.001
eGFR_MDRD_						
log⁡(Neck circumference)	0.923	13.96 (14.05)	0.322	0.945	−7.1 (20.16)	0.726
log⁡(Body mass index)	0.923	−11.03 (8.88)	0.217	0.945	3.87 (12.42)	0.757
log⁡(Waist circumference)	0.922	9.6 (11.86)	0.420	0.946	12.38 (14.28)	0.391
log⁡(Hip circumference)	0.923	20.57 (16.24)	0.208	0.946	−14.26 (19.12)	0.460
log⁡(Cystatin C)						
log⁡(Neck circumference)	0.674	0.33 (0.2)	0.098	0.619	0.14 (0.34)	0.681
log⁡(Body mass index)	0.665	0.02 (0.12)	0.852	0.639	0.3 (0.2)	0.141
log⁡(Waist circumference)	0.670	0.21 (0.17)	0.215	0.637	0.33 (0.23)	0.169
log⁡(Hip circumference)	0.679	0.47 (0.22)	0.037	0.631	0.36 (0.3)	0.242
Uric Acid						
log⁡(Neck circumference)	0.138	−3.49 (3.74)	0.353	0.346	17.8 (5.5)	0.002
log⁡(Body mass index)	0.133	1.17 (2.3)	0.612	0.319	9.93 (3.46)	0.006
log⁡(Waist circumference)	0.132	0.87 (3.08)	0.778	0.262	8.4 (4.17)	0.050
log⁡(Hip circumference)	0.136	−3.34 (4.24)	0.432	0.296	13.84 (5.44)	0.014
log⁡(Urine microalbuminuria)						
log⁡(Neck circumference)	0.096	2.62 (1.46)	0.075	0.271	6.68 (2.1)	0.003
log⁡(Body mass index)	0.168	3.24 (0.89)	<0.001	0.168	2.47 (1.36)	0.077
log⁡(Waist circumference)	0.142	3.64 (1.18)	0.003	0.191	3.35 (1.56)	0.037
log⁡(Hip circumference)	0.134	4.74 (1.64)	0.005	0.145	3.02 (2.14)	0.164
log⁡(hsCRP)						
log⁡(Neck circumference)	0.073	3 (1.39)	0.033	0.114	4.25 (1.97)	0.037
log⁡(Body mass index)	0.094	2.3 (0.85)	0.008	0.072	1.92 (1.24)	0.127
log⁡(Waist circumference)	0.085	2.85 (1.13)	0.014	0.074	2.26 (1.43)	0.121
log⁡(Hip circumference)	0.073	3.44 (1.58)	0.031	0.104	3.82 (1.88)	0.048
log⁡(Systolic blood pressure)						
log⁡(Neck circumference)	0.017	0.14 (0.12)	0.248	0.028	0.14 (0.17)	0.388
log⁡(Body mass index)	0.045	0.16 (0.07)	0.030	0.012	0.01 (0.1)	0.927
log⁡(Waist circumference)	0.034	0.19 (0.1)	0.066	0.022	0.08 (0.12)	0.494
log⁡(Hip circumference)	0.037	0.27 (0.14)	0.053	0.017	0.08 (0.16)	0.633
Diastolic blood pressure						
log⁡(Neck circumference)	0.127	27.95 (19.83)	0.161	0.211	29.04 (29.34)	0.328
log⁡(Body mass index)	0.169	35.02 (12.26)	0.005	0.197	8.09 (18.23)	0.659
log⁡(Waist circumference)	0.132	27.86 (16.66)	0.097	0.227	28.83 (20.72)	0.171
log⁡(Hip circumference)	0.139	43.71 (22.83)	0.058	0.240	45.54 (27.46)	0.104
log⁡(Fasting plasma glucose)						
log⁡(Neck circumference)	0.033	0.47 (0.29)	0.103	0.068	0.09 (0.41)	0.826
log⁡(Body mass index)	0.061	0.45 (0.18)	0.014	0.068	0.08 (0.25)	0.765
log⁡(Waist circumference)	0.100	0.8 (0.23)	0.001	0.069	0.1 (0.29)	0.733
log⁡(Hip circumference)	0.041	0.64 (0.33)	0.055	0.077	−0.28 (0.38)	0.469
log⁡(Triglyceride)						
log⁡(Neck circumference)	0.138	1.49 (0.46)	0.002	0.436	2.25 (0.69)	0.002
log⁡(Body mass index)	0.090	0.57 (0.3)	0.061	0.355	0.92 (0.47)	0.056
log⁡(Waist circumference)	0.088	0.74 (0.4)	0.068	0.384	1.27 (0.52)	0.018
log⁡(Hip circumference)	0.095	1.14 (0.55)	0.040	0.351	1.32 (0.7)	0.067
Total cholesterol						
log⁡(Neck circumference)	0.033	133.61 (85.5)	0.121	0.007	−71.5 (156.05)	0.649
log⁡(Body mass index)	0.023	59.89 (54.46)	0.274	0.004	22.75 (96.28)	0.814
log⁡(Waist circumference)	0.021	69.07 (72.53)	0.343	0.006	45.77 (111.37)	0.683
log⁡(Hip circumference)	0.016	59 (99.97)	0.556	0.003	29.37 (149.02)	0.845
LDL cholesterol						
log⁡(Neck circumference)	0.048	157.85 (72.6)	0.032	0.012	28.43 (143.31)	0.844
log⁡(Body mass index)	0.052	105.66 (45.9)	0.023	0.033	88.74 (87.34)	0.315
log⁡(Waist circumference)	0.029	94.18 (61.81)	0.130	0.017	54.16 (101.96)	0.598
log⁡(Hip circumference)	0.016	75.3 (85.54)	0.381	0.017	69.33 (136.28)	0.613
log⁡(HDL cholesterol)						
log⁡(Neck circumference)	0.023	−0.2 (0.29)	0.494	0.202	−0.86 (0.4)	0.039
log⁡(Body mass index)	0.051	−0.36 (0.18)	0.049	0.123	−0.07 (0.26)	0.782
log⁡(Waist circumference)	0.028	−0.25 (0.24)	0.305	0.128	−0.18 (0.3)	0.562
log⁡(Hip circumference)	0.022	−0.18 (0.34)	0.596	0.124	−0.15 (0.41)	0.724

*R* ^2^: unadjusted *R* ^2^; *B*: unstandardized coefficients beta; SE: unstandardized coefficients Std. Error.
